# Supplementary material for: Distribution and evolution of the western European water frogs (genus Pelophylax) from Catalonia, northeastern Spain
Source: PeerJ. 2025 Sep 22;13:e19895. doi: 10.7717/peerj.19895 (PMC12462689; doi:10.7717/peerj.19895)
Supplement: Supplemental Information 2 — Haplotype designation is assigned for the specimens sequenced in this study (see Table S1) [file peerj-13-19895-s002.docx]

**Table S2**

List of the specimens used for the cytb phylogenetic analyses. Haplotype designation is assigned for the specimens sequenced in this study (see Table S1).

| **Code** | **Species** | **Haplotype** | **Locality** | **Accession No.** | **Reference** |  |
| --- | --- | --- | --- | --- | --- | --- |
| CN11457 | *P.* kl. *grafi* | H1 | Catalonia, Mas de Barberans | PQ871183 | This study |  |
| CN12373 | *P.* kl. *grafi* | H2 | Catalonia, Pals | PQ871184 | This study |  |
| CN12190 | *P.* kl. *grafi* | H3 | Catalonia, Parets del Vallès | PQ871185 | This study |  |
| CN12177 | *P.* kl. *grafi* | H4 | Catalonia, Pals | PQ871186 | This study |  |
| CN13058 | *P.* kl. *grafi* | H5 | Catalonia, Esponellà | PQ871187 | This study |  |
| CN13319 | *P.* kl. *grafi* | H6 | Catalonia, Solsona | PQ871188 | This study |  |
| CN12981 | *P.* kl. *grafi* | H7 | Catalonia, Sant Llorenç de la Muga | PQ871189 | This study |  |
| CN12118 | *P.* kl. *grafi* | H8 | Catalonia, Pals | PQ871190 | This study |  |
| CN13053 | *P.* kl. *grafi* | H9 | Catalonia, Esponellà | PQ871191 | This study |  |
| CN12950 | *P.* kl. *grafi* | H10 | Catalonia, Lleida | PQ871192 | This study |  |
| CN12126 | *P.* kl. *grafi* | H11 | Catalonia, Aiguamolls de l'Empordà | PQ871193 | This study |  |
| CN12463 | *P.* kl. *grafi* | H12 | Catalonia, Castellcir | PQ871194 | This study |  |
| CN12109 | *P.* kl. *grafi* | H13 | Catalonia, Aiguamolls de l'Empordà | PQ871195 | This study |  |
| CN13442 | *P.* kl. *grafi* | H14 | Catalonia, Parc Natural de Sant Llorenç del Munt i l'Obac | PQ871196 | This study |  |
| CN12169 | *P.* kl. *grafi* | H15 | Catalonia, Albera | PQ871197 | This study |  |
| CN12503 | *P.* kl. *grafi* | H16 | Catalonia, Parc del Montnegre i el Corredor | PQ871198 | This study |  |
| CN13100 | *P.* kl. *grafi* | H17 | Catalonia, Esponellà | PQ871199 | This study |  |
| CN11569 | *P.* kl. *grafi* | H18 | Catalonia, Mas de Barberans | PQ871200 | This study |  |
| CN11468 | *P.* kl. *grafi* | H19 | Catalonia, Parc Natural del Delta de l'Ebre, Poblenou del Delta | PQ871201 | This study |  |
| CN11477 | *P.* kl. *grafi* | H20 | Catalonia, Pals | PQ871202 | This study |  |
| CN13046 | *P.* kl. *grafi* | H21 | Catalonia, Esponellà | PQ871203 | This study |  |
| CN11540 | *P.* kl. *grafi* | H22 | Catalonia, Parc d'Olèrdola | PQ871204 | This study |  |
| PER01 | *P. perezi* |  | Spain | MF667559 | Dufresnes et al. (2017) |  |
| PER04 | *P. perezi* |  | Spain | MF667566 | Dufresnes et al. (2017) |  |
| PER05 | *P. perezi* |  | Spain | MF667563 | Dufresnes et al. (2017) |  |
| PER06 | *P. perezi* |  | Spain, Gran Canaria | MF667561 | Dufresnes et al. (2017) |  |
| PER07 | *P. perezi* |  | Spain, Trujillo | MF667562 | Dufresnes et al. (2017) |  |
| PER09 | *P. perezi* |  | France, Penchant du Salagou | MF667571 | Dufresnes et al. (2017) |  |
| PER10 | *P. perezi* |  | France, Lac de Condamine | MF667568 | Dufresnes et al. (2017) |  |
| PER11 | *P. perezi* |  | France, Lac de Condamine | MF667567 | Dufresnes et al. (2017) |  |
| PER12 | *P. perezi* |  | France, Ville Vieille | MF667576 | Dufresnes et al. (2017) |  |
| PER13 | *P. perezi* |  | France, Octon (Plage-Salagou) | MF667574 | Dufresnes et al. (2017) |  |
| PER14 | *P. perezi* |  | France, Penchant du Salagou | MF667575 | Dufresnes et al. (2017) |  |
| PER15 | *P. perezi* |  | France, Source du Goutal (Trop plein) | MF667569 | Dufresnes et al. (2017) |  |
| PER17 | *P. perezi* |  | France, Octon (Plage-Salagou) | MF667572 | Dufresnes et al. (2017) | |
| PER18 | *P. perezi* |  | France, Devois la Trivalle | MF667573 | Dufresnes et al. (2017) |  |
| Rper | *P. perezi* |  | France | AY043052 | Lymberakis et al. (2007) |  |
| Rsah1 | *P. saharicus* |  | Tunis, Ickeul Mt. | DQ474177 | Lymberakis et al. (2007) |  |
| Rles1 | *P. lessonae* |  | Belgium | AY057099 | Lymberakis et al. (2007) |  |
| Rrid3 | *P. ridibundus* |  | Greece, Thrace (Therma) | DQ474162 | Lymberakis et al. (2007) |  |
| Rkurt4 | *P. kurtmuelleri* |  | Greece, Prespa L. | DQ474159 | Lymberakis et al. (2007) |  |
| Rbed11 | *P. bedriagae* |  | Greece, Macedonia (Dadia) | DQ474139 | Lymberakis et al. (2007) |  |
| Rep1 | *P. epeirotica* |  | Greece, Peloponnesos (Lisimaxia L.) | DQ474153 | Lymberakis et al. (2007) |  |
| Rcret6 | *P. cretensis* |  | Greece, Crete Isl. (Kaloudiana L.) | DQ474150 | Lymberakis et al. (2007) |  |
| Rcat | *L. catesbeianus* |  | Greece, Crete Isl. | DQ474180 | Lymberakis et al. (2007) |  |
