# Supplementary material for: Distribution and evolution of the western European water frogs (genus Pelophylax) from Catalonia, northeastern Spain
Source: PeerJ. 2025 Sep 22;13:e19895. doi: 10.7717/peerj.19895 (PMC12462689; doi:10.7717/peerj.19895)
Supplement: Supplemental Information 3 [file peerj-13-19895-s003.pdf]

Figure S1. Gel image showing results on the two-step molecular identification protocol. A) Gel showing the digestion pattern of the marker RAG1 with the restriction enzyme EcoO109I. The first group (*P. perezi*, *P. lessonae* and *P. bergeri*) have no cleavage site for the restriction enzyme, resulting in a band of 1,000 bp, which represents the whole marker sequence. The second group (*P. ridibundus* and *P. kurtmuelleri*) has one cleavage site and produces two bands of around 400 bp and 600 bp. The species of the third group (*P.* kl. *grafi*, *P.* kl. *esculentus*, *P.* kl. *hispanicus* and klepton *PK*) have two different genomes, one from the first group which is represented by a band of 1,000 bp; the other belonging to a species from the second group, which produces two faint bands of 400 bp and 600 bp. B) Gel image showing size differences of the SAI-1 PCR product for *P. perezi* (853 pb), *P. lessonae* (306 pb), *P. bergeri* (298 bp), *P. ridibundus* (839-843 pb*)* and *P. kurtmuelleri* (717 bp). Kleptons contain two bands, corresponding to the sizes of their respective parental species. Kleptons PK, *P.* kl. *hispanicus* and *P.* kl.*esculentus* were obtained in silico by mixing DNA from the parental species.

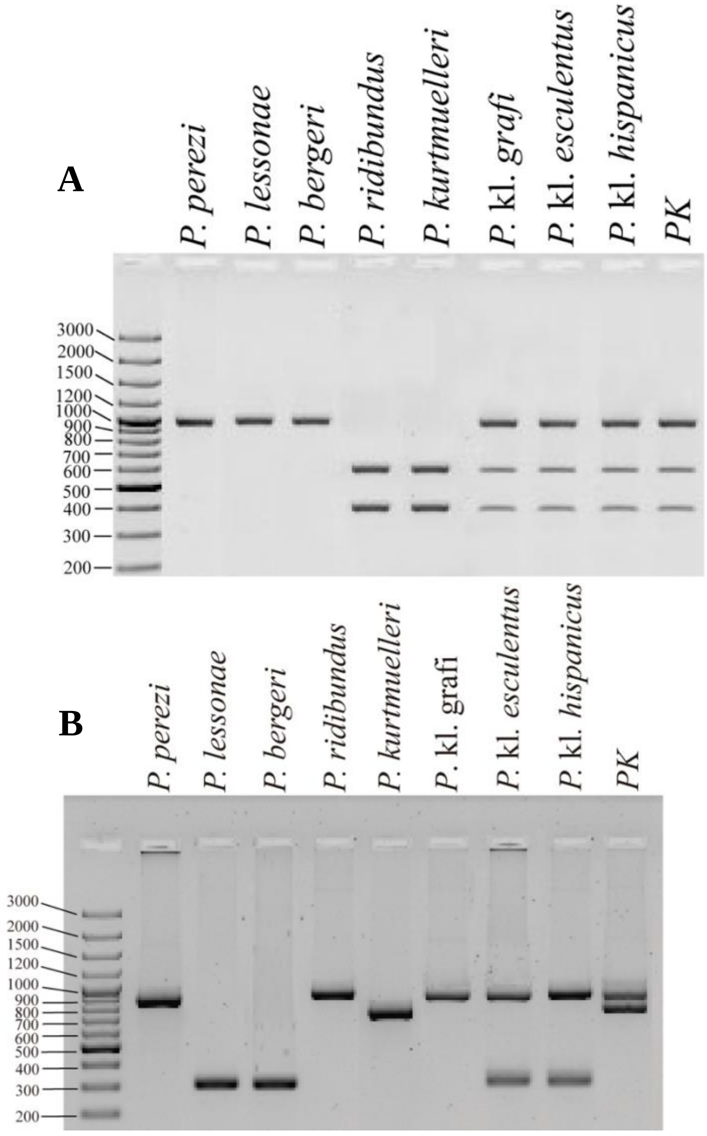

Figure S2. Results of the first step of the two-step molecular identification protocol for European water frogs. Agarose gels display the product of RAG1 molecular marker digestion using the EcoO109I restriction enzyme.

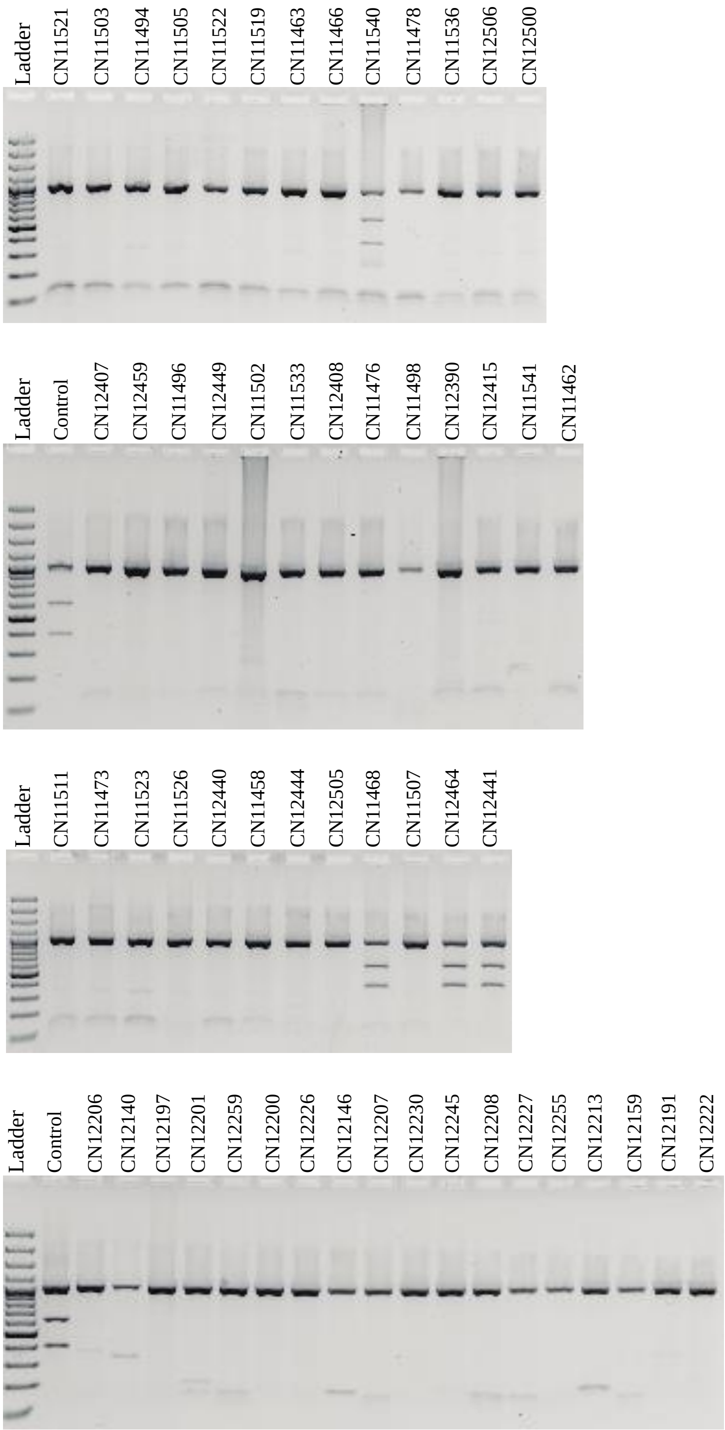

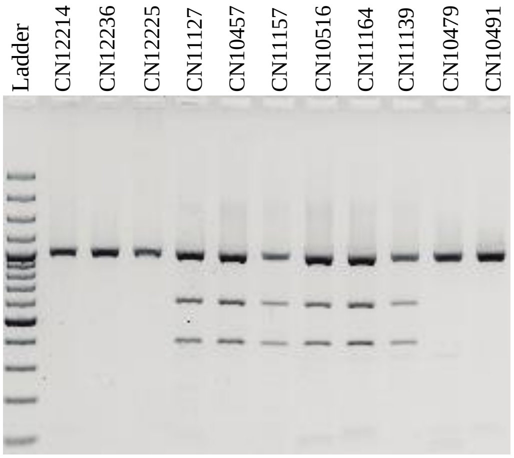
Ladder
CN12214
CN12236
CN12225
CN11127
CN10457
CN11157
CN10516
CN11164
CN11139
CN10479
CN10491

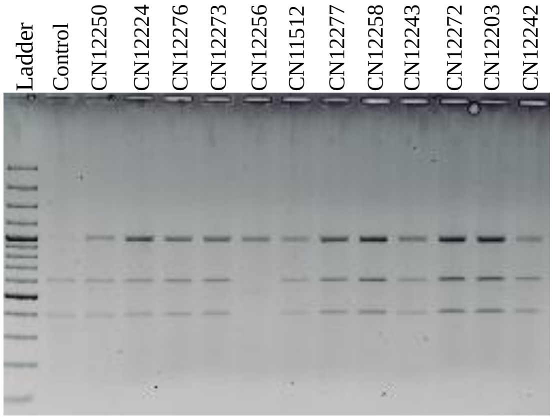
Ladder
Control
CN12250
CN12224
CN12276
CN12273
CN12256
CN11512
CN12277
CN12258
CN12243
CN12272
CN12203
CN12242

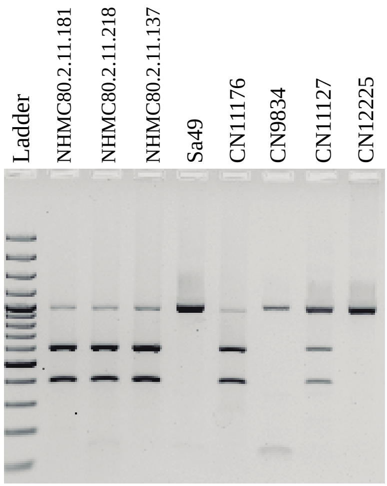
Ladder
NHMC80.2.11.181
NHMC80.2.11.218
NHMC80.2.11.137
Sa49
CN11176
CN9834
CN11127
CN12225

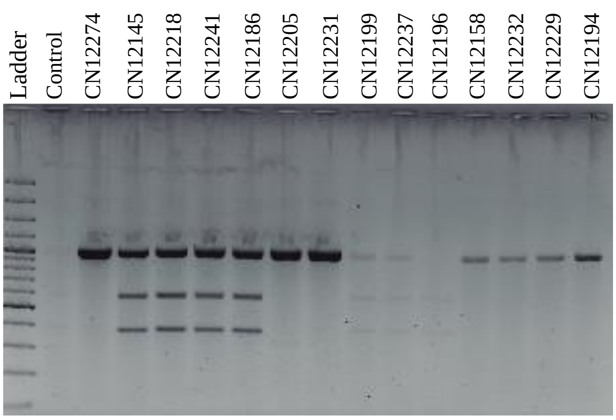
Ladder
Control
CN12274
CN12145
CN12218
CN12241
CN12186
CN12205
CN12231
CN12199
CN12237
CN12196
CN12158
CN12232
CN12229
CN12194

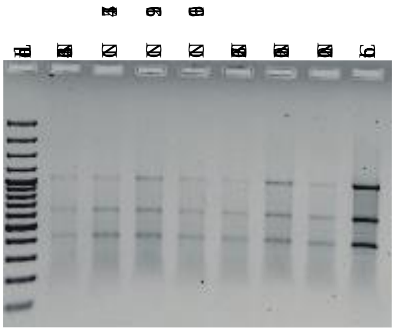

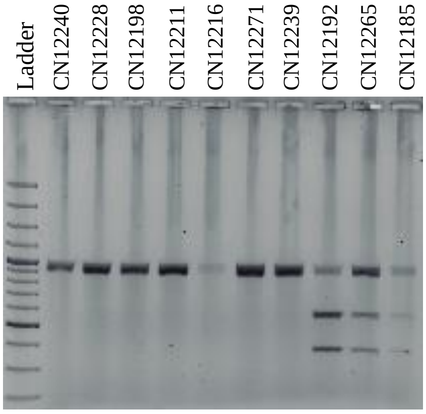
Ladder
CN12240
CN12228
CN12198
CN12211
CN12216
CN12271
CN12239
CN12192
CN12265
CN12185

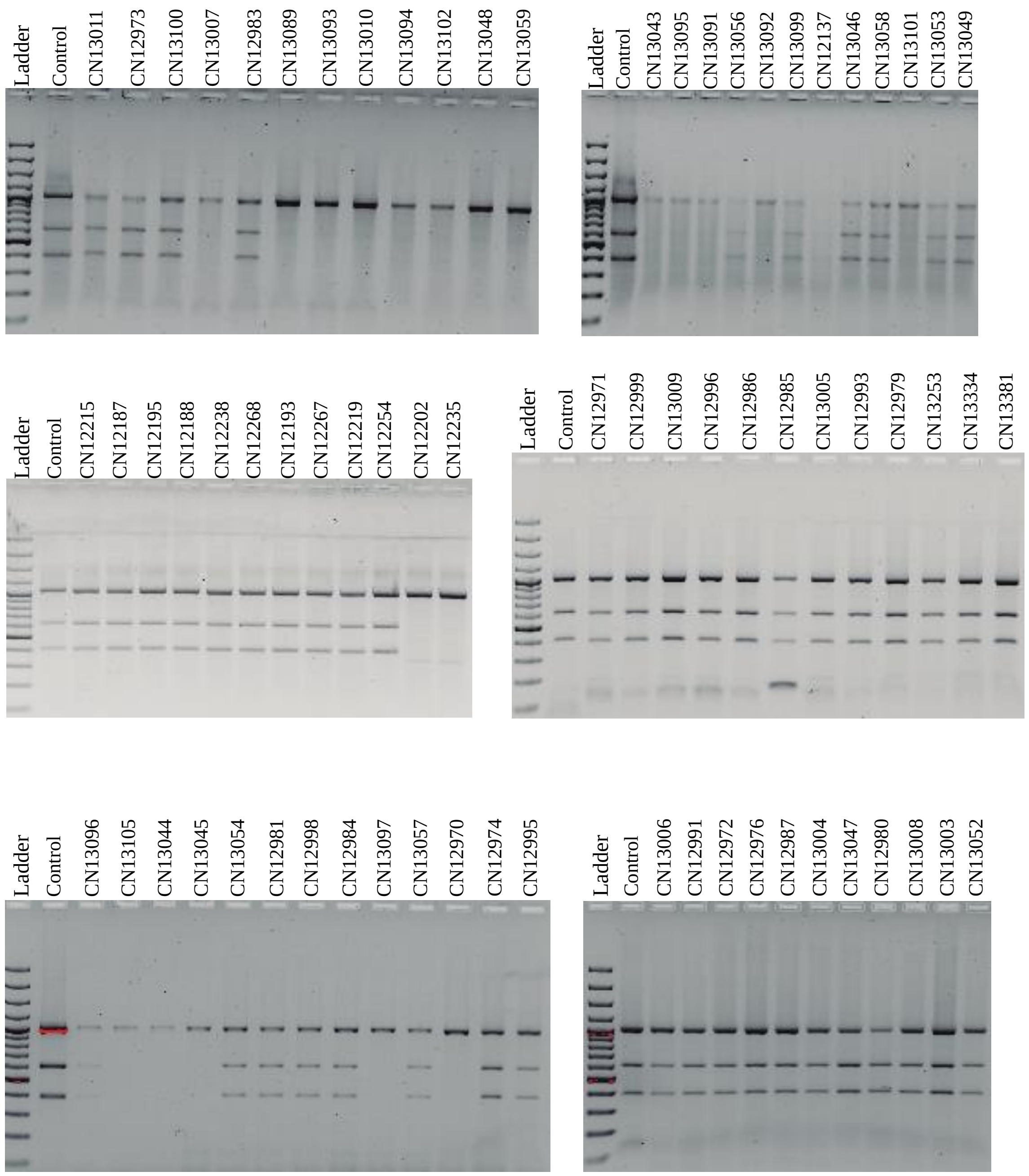
Ladder
Control
CN13011
CN12973
CN13100
CN13007
CN12983
CN13089
CN13093
CN13010
CN13094
CN13102
CN13048
CN13059
Ladder
Control
CN13043
CN13095
CN13091
CN13056
CN13092
CN13099
CN12137
CN13046
CN13058
CN13101
CN13053
CN13049
Ladder
Control
CN12215
CN12187
CN12195
CN12188
CN12238
CN12268
CN12193
CN12267
CN12219
CN12254
CN12202
CN12235
Ladder
Control
CN12971
CN12999
CN13009
CN12996
CN12986
CN12985
CN13005
CN12993
CN12979
CN13253
CN13334
CN13381
Ladder
Control
CN13096
CN13105
CN13044
CN13045
CN13054
CN12981
CN12998
CN12984
CN13097
CN13057
CN12970
CN12974
CN12995
Ladder
Control
CN13006
CN12991
CN12972
CN12976
CN12987
CN13004
CN13047
CN12980
CN13008
CN13003
CN13052

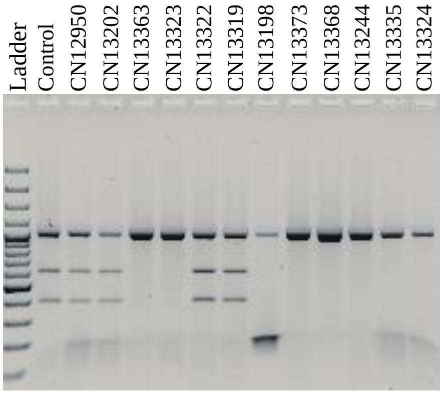
Ladder
Control
CN12950
CN13202
CN13363
CN13323
CN13322
CN13319
CN13198
CN13373
CN13368
CN13244
CN13335
CN13324

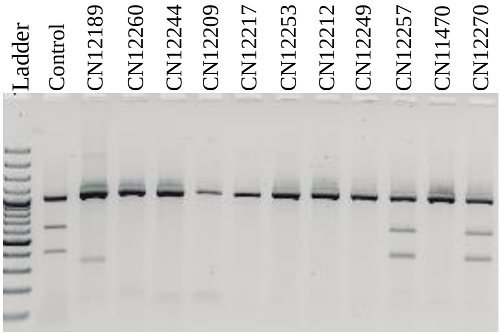
Ladder
Control
CN12189
CN12260
CN12244
CN12209
CN12217
CN12253
CN12212
CN12249
CN12257
CN11470
CN12270

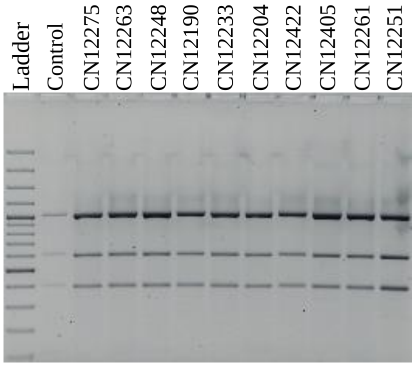
Ladder
Control
CN12275
CN12263
CN12248
CN12190
CN12233
CN12204
CN12422
CN12405
CN12261
CN12251

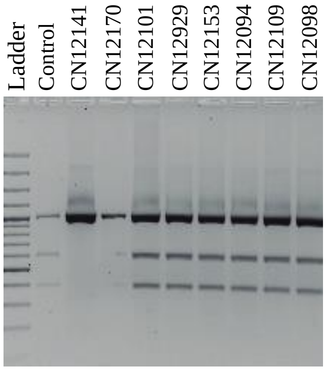
Ladder
Control
CN12141
CN12170
CN12101
CN12929
CN12153
CN12094
CN12109
CN12098

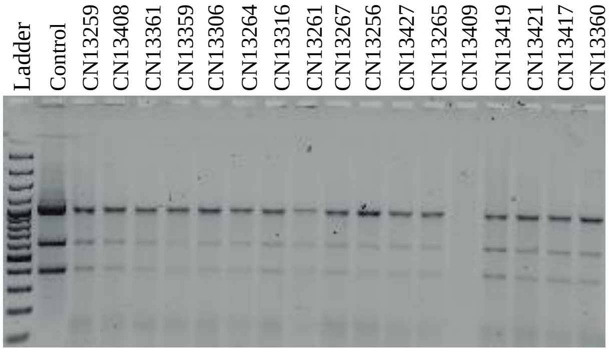
Ladder
Control
CN13259
CN13408
CN13361
CN13359
CN13306
CN13264
CN13316
CN13261
CN13267
CN13256
CN13427
CN13265
CN13409
CN13419
CN13421
CN13417
CN13360

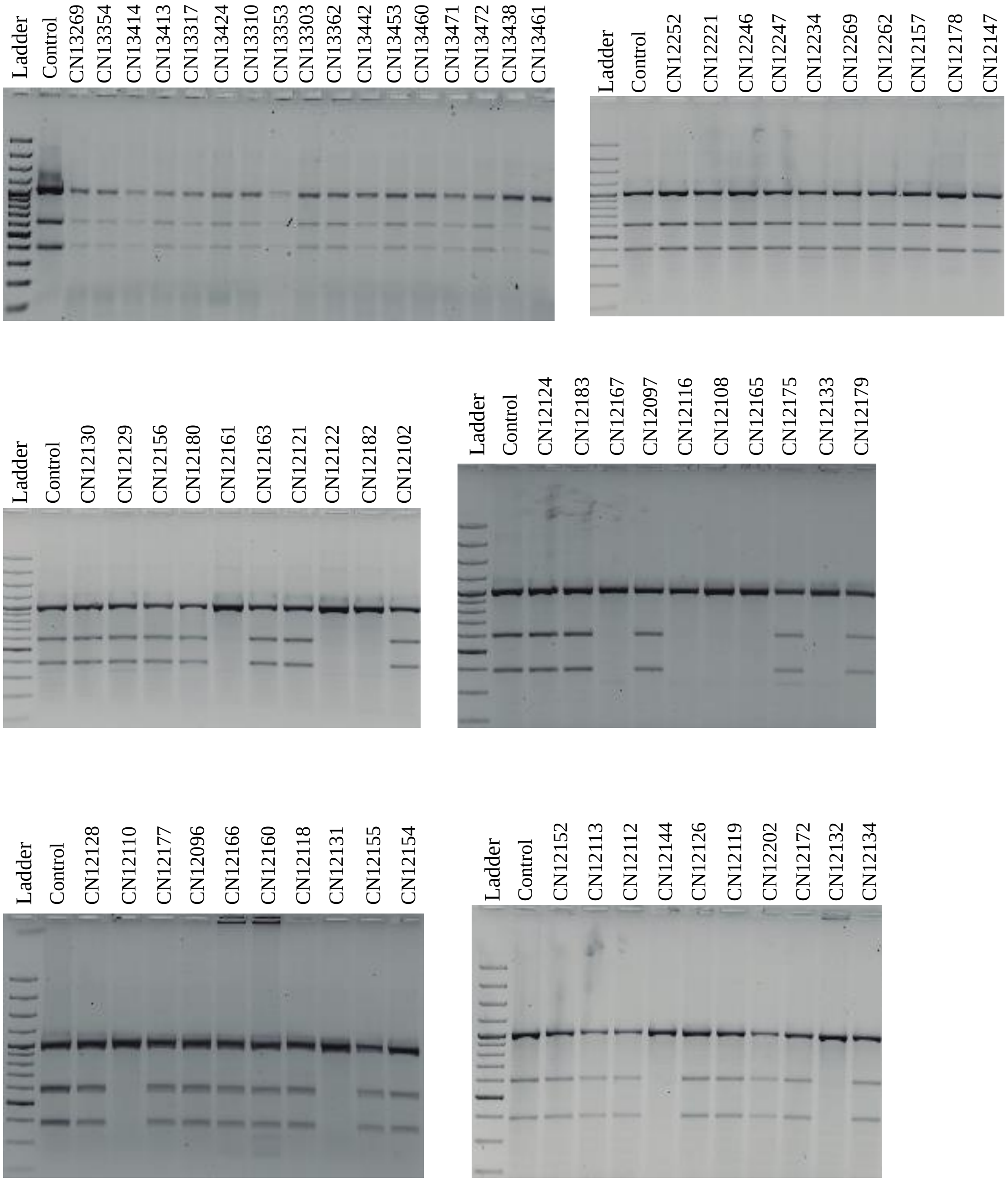
Ladder
Control
CN13269
CN13354
CN13414
CN13413
CN13317
CN13424
CN13310
CN13353
CN13303
CN13362
CN13442
CN13453
CN13460
CN13471
CN13472
CN13438
CN13461
Ladder
Control
CN12252
CN12221
CN12246
CN12247
CN12234
CN12269
CN12262
CN12157
CN12178
CN12147
Ladder
Control
CN12130
CN12129
CN12156
CN12180
CN12161
CN12163
CN12121
CN12122
CN12182
CN12102
Ladder
Control
CN12124
CN12183
CN12167
CN12097
CN12116
CN12108
CN12165
CN12175
CN12133
CN12179
Ladder
Control
CN12128
CN12110
CN12177
CN12096
CN12166
CN12160
CN12118
CN12131
CN12155
CN12154
Ladder
Control
CN12152
CN12113
CN12112
CN12144
CN12126
CN12119
CN12202
CN12172
CN12132
CN12134

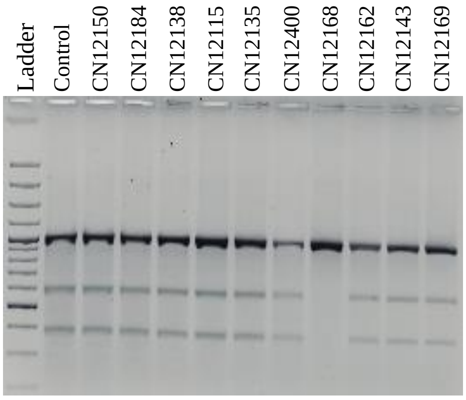
Ladder
Control
CN12150
CN12184
CN12138
CN12115
CN12135
CN12400
CN12168
CN12162
CN12143
CN12169

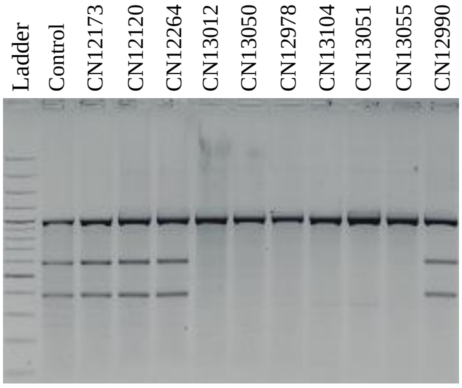
Ladder
Control
CN12173
CN12120
CN12264
CN13012
CN13050
CN12978
CN13104
CN13051
CN13055
CN12990

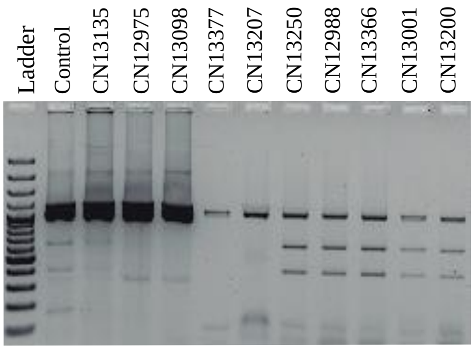
Ladder
Control
CN13135
CN12975
CN13098
CN13377
CN13207
CN13250
CN12988
CN13366
CN13001
CN13200

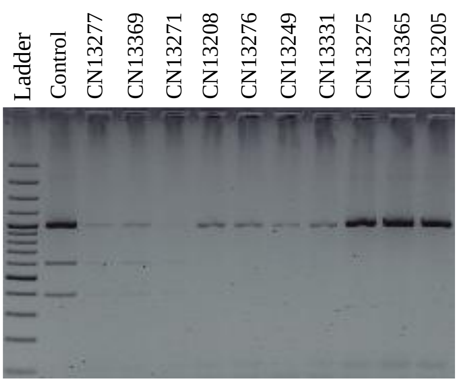
Ladder
Control
CN13277
CN13369
CN13271
CN13208
CN13276
CN13249
CN13331
CN13275
CN13365
CN13205

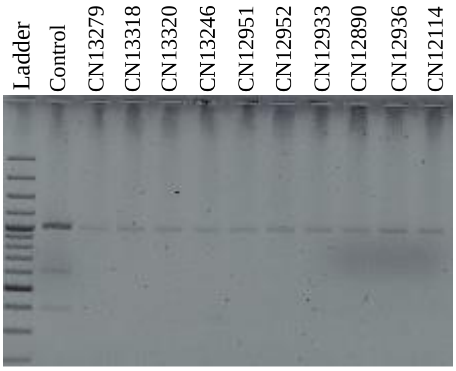
Ladder
Control
CN13279
CN13318
CN13320
CN13246
CN12951
CN12952
CN12933
CN12890
CN12936
CN12114

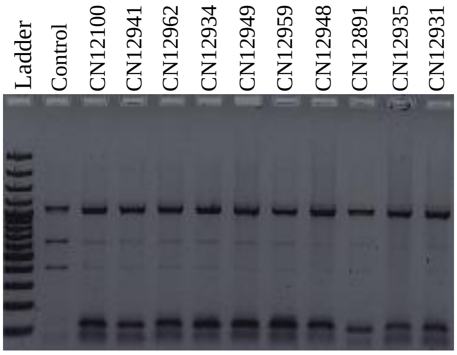
Ladder
Control
CN12100
CN12941
CN12962
CN12934
CN12949
CN12959
CN12948
CN12891
CN12935
CN12931

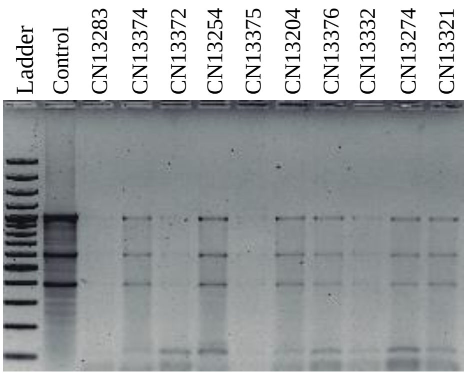
Ladder
Control
CN13283
CN13374
CN13372
CN13254
CN13375
CN13204
CN13376
CN13332
CN13274
CN13321

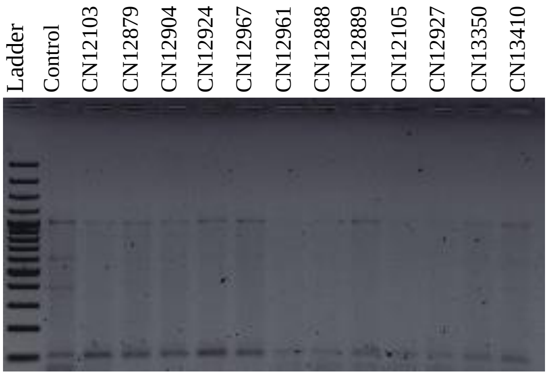
Ladder
Control
CN12103
CN12879
CN12904
CN12924
CN12967
CN12961
CN12888
CN12889
CN12105
CN12927
CN13350
CN13410

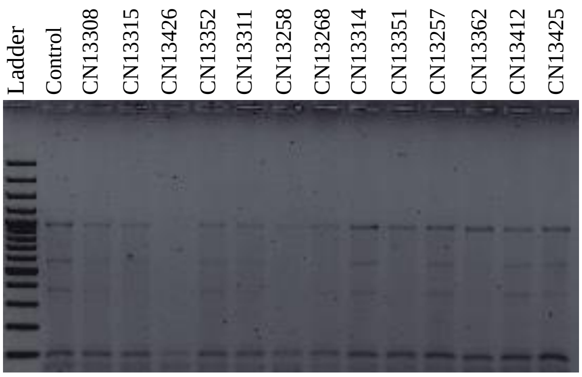
Ladder
Control
CN13308
CN13315
CN13426
CN13352
CN13311
CN13258
CN13268
CN13314
CN13351
CN13257
CN13362
CN13412
CN13425

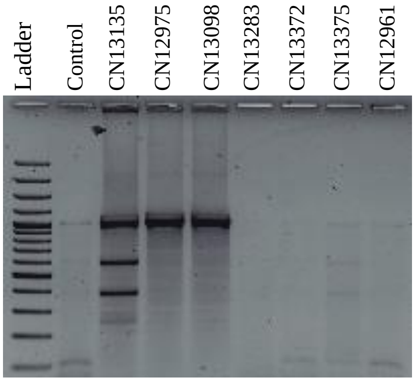
Ladder
Control
CN13135
CN12975
CN13098
CN13283
CN13372
CN13375
CN12961

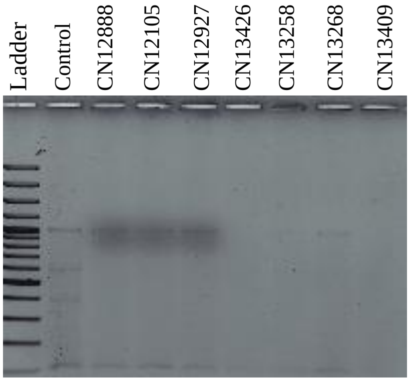
Ladder
Control
CN12888
CN12105
CN12927
CN13426
CN13258
CN13268
CN13409

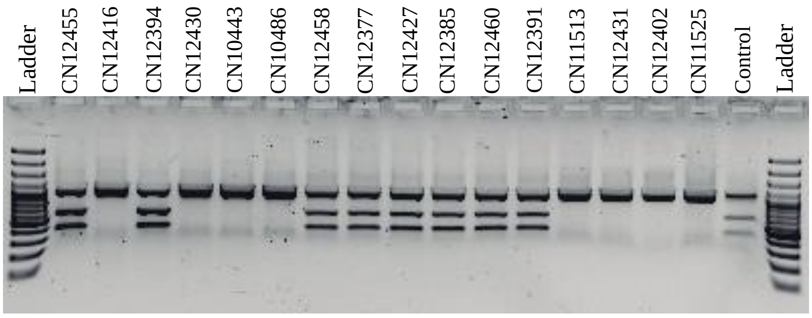
Ladder
CN12455
CN12416
CN12394
CN12430
CN10443
CN10486
CN12458
CN12377
CN12427
CN12385
CN12460
CN12391
CN11513
CN12431
CN12402
CN11525
Control
Ladder

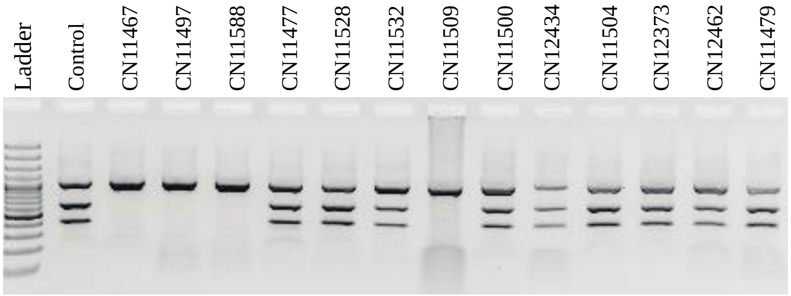
Ladder
Control
CN11467
CN11497
CN11588
CN11477
CN11528
CN11532
CN11509
CN11500
CN12434
CN11504
CN12373
CN12462
CN11479

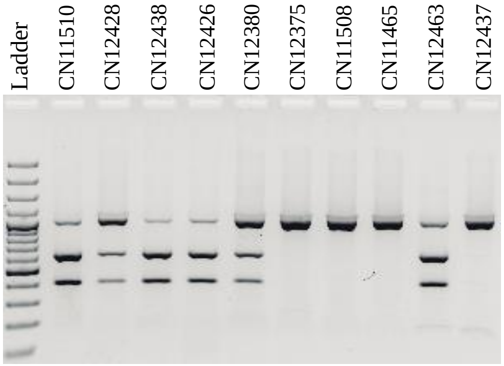
Ladder
CN11510
CN12428
CN12438
CN12426
CN12380
CN12375
CN11508
CN11465
CN12463
CN12437

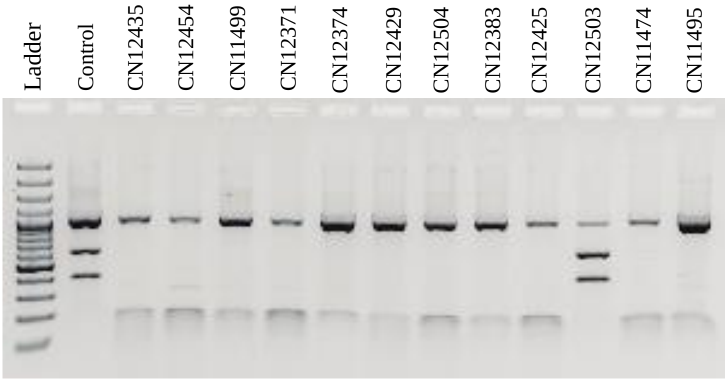
Ladder
Control
CN12435
CN12454
CN11499
CN12371
CN12374
CN12429
CN12504
CN12383
CN12425
CN12503
CN11474
CN11495

Figure S3. Results of the second step of the two-step molecular identification protocol for European water frogs. Agarose gels display the SAI-1 PCR product amplified for all the samples collected in this study.

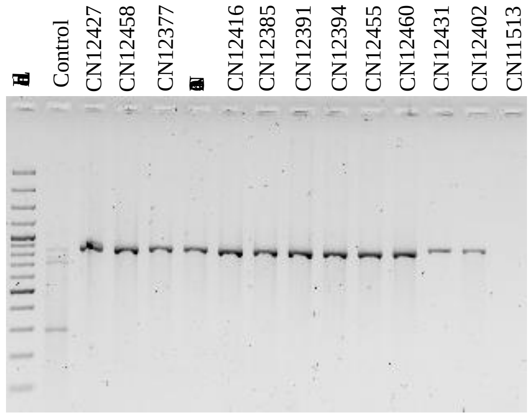

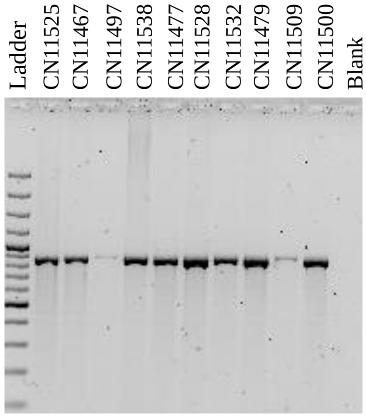

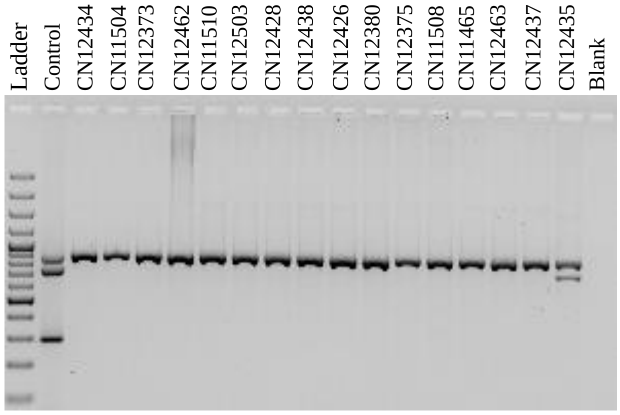

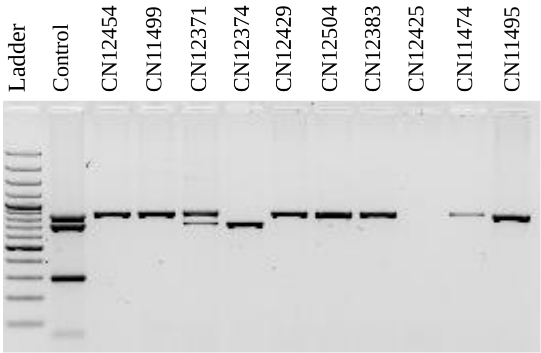
Ladder
Control
CN12454
CN11499
CN12371
CN12374
CN12429
CN12504
CN12383
CN12425
CN11474
CN11495

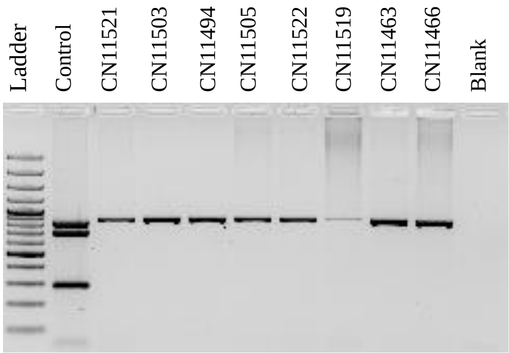
Ladder
Control
CN11521
CN11503
CN11494
CN11505
CN11522
CN11519
CN11463
CN11466
Blank

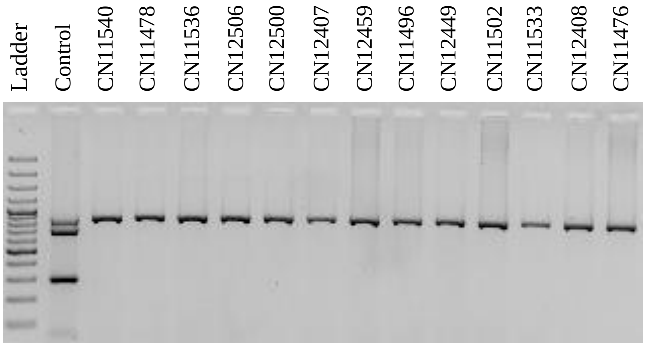
Ladder
Control
CN11540
CN11478
CN11536
CN12506
CN12500
CN12407
CN12459
CN11496
CN12449
CN11502
CN11533
CN12408
CN11476

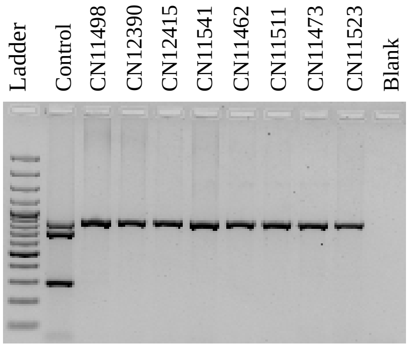
Ladder
Control
CN11498
CN12390
CN12415
CN11541
CN11462
CN11511
CN11473
CN11523
Blank

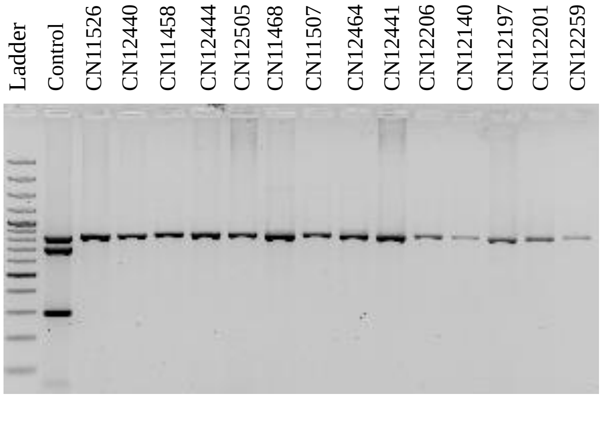
Ladder
Control
CN11526
CN12440
CN11458
CN12444
CN12505
CN11468
CN11507
CN12464
CN12441
CN12206
CN12140
CN12197
CN12201
CN12259

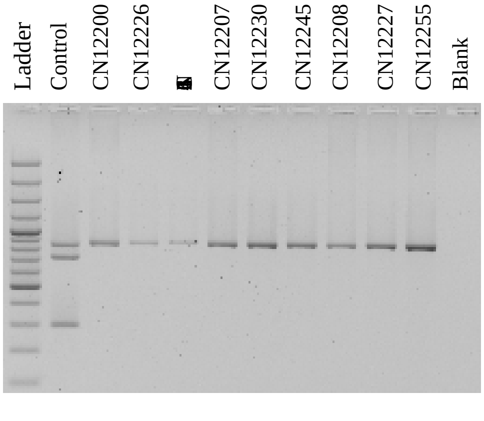
Ladder
Control
CN12200
CN12226
CN12207
CN12230
CN12245
CN12208
CN12227
CN12255
Blank

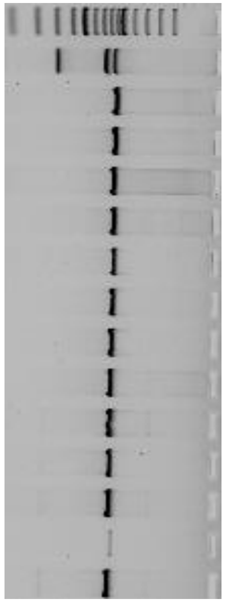
Ladder
Control
CN12213
CN12159
CN12191
CN12222
CN12214
CN12236
CN12225
CN11127
CN10457
CN11126
CN11157
CN10516
CN11164

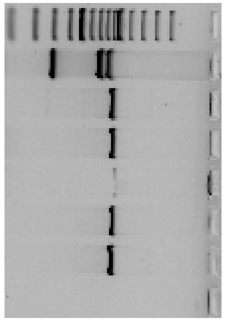
Ladder
Control
CN11139
CN10443
CN10486
CN10479
CN10491
Blank

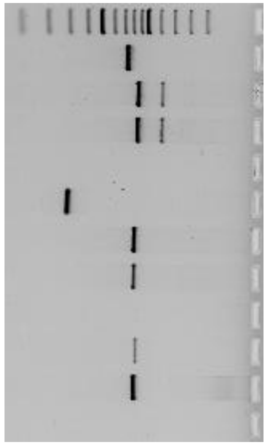
Ladder
NHMC80.2.11.181
NHMC80.2.11.218
NHMC80.2.11.137
S47
Sa49
CN11176
CN11175
CN12141
CN12170
CN12101
Blank

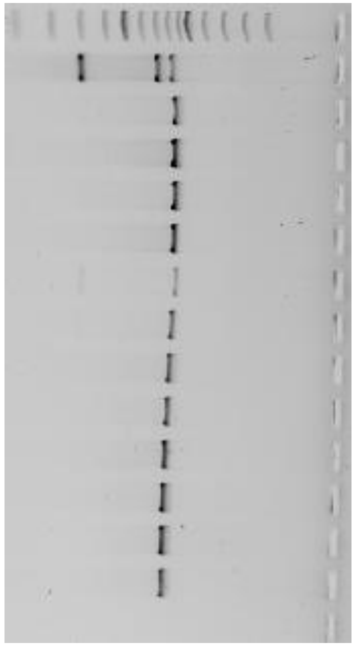
Ladder
Control
CN12250
CN12224
CN12276
CN12273
CN12256
CN11512
CN12277
CN12258
CN12243
CN12272
CN12203
CN12242
Blank

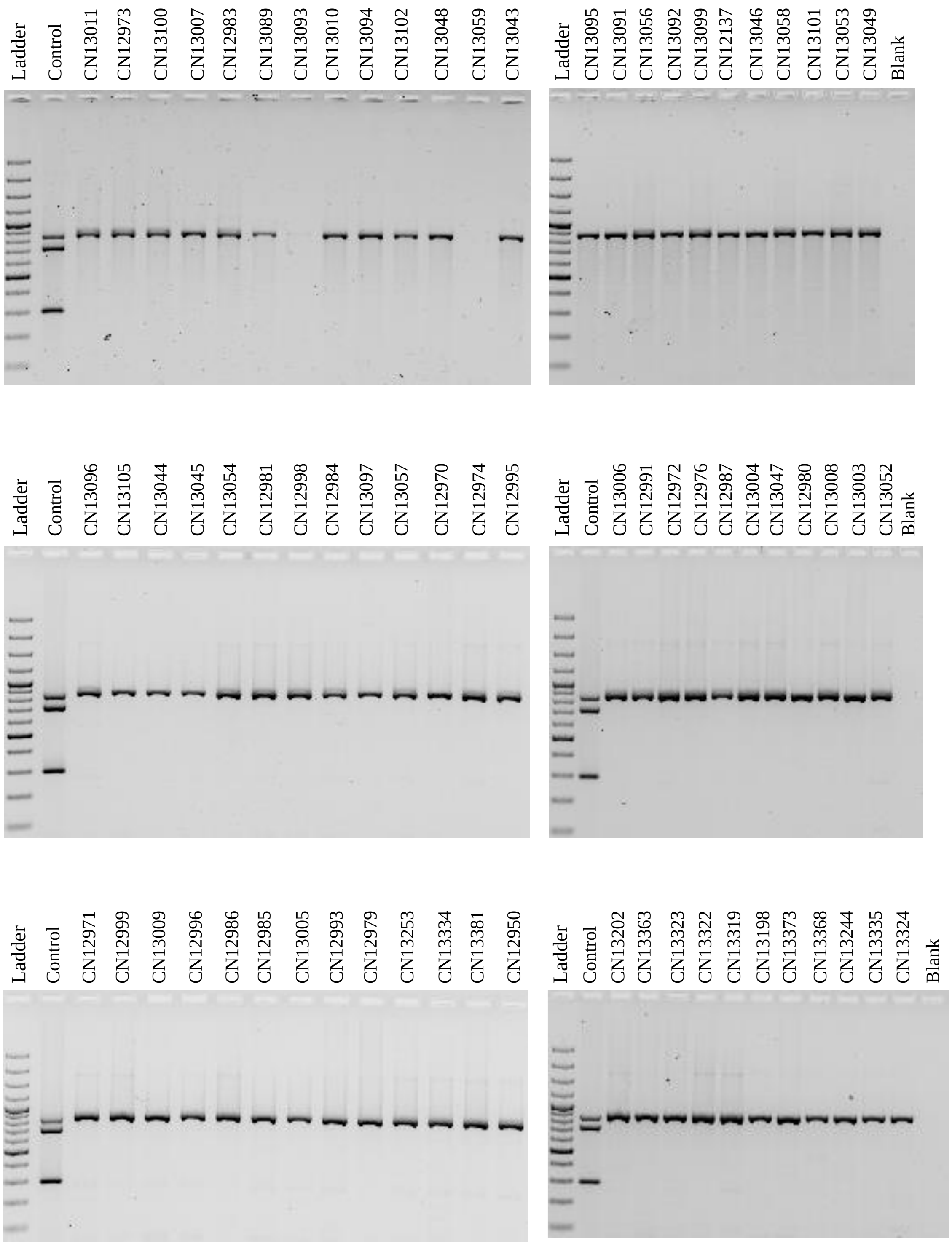
Ladder
Control
CN13011
CN12973
CN13100
CN13007
CN12983
CN13089
CN13093
CN13010
CN13094
CN13102
CN13048
CN13059
CN13043
Ladder
CN13095
CN13091
CN13056
CN13092
CN13099
CN12137
CN13046
CN13058
CN13101
CN13053
CN13049
Blank
Ladder
Control
CN13096
CN13105
CN13044
CN13045
CN13054
CN12981
CN12998
CN12984
CN13097
CN13057
CN12970
CN12974
CN12995
Ladder
Control
CN13006
CN12991
CN12972
CN12976
CN12987
CN13004
CN13047
CN12980
CN13008
CN13003
CN13052
Blank
Ladder
Control
CN12971
CN12999
CN13009
CN12996
CN12986
CN12985
CN13005
CN12993
CN12979
CN13253
CN13334
CN13381
CN12950
Ladder
Control
CN13202
CN13363
CN13323
CN13322
CN13319
CN13198
CN13373
CN13368
CN13244
CN13335
CN13324
Blank

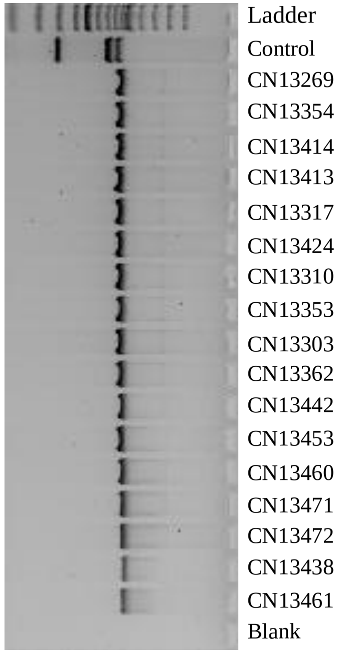
Ladder
Control
CN13269
CN13354
CN13414
CN13413
CN13317
CN13424
CN13310
CN13353
CN13303
CN13362
CN13442
CN13453
CN13460
CN13471
CN13472
CN13438
CN13461
Blank

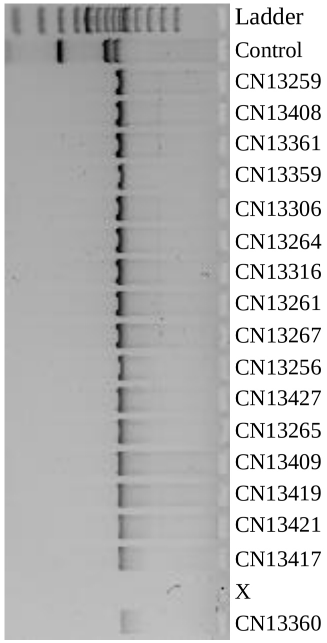
Ladder
Control
CN13259
CN13408
CN13361
CN13359
CN13306
CN13264
CN13316
CN13261
CN13267
CN13256
CN13427
CN13265
CN13409
CN13419
CN13421
CN13417
X
CN13360

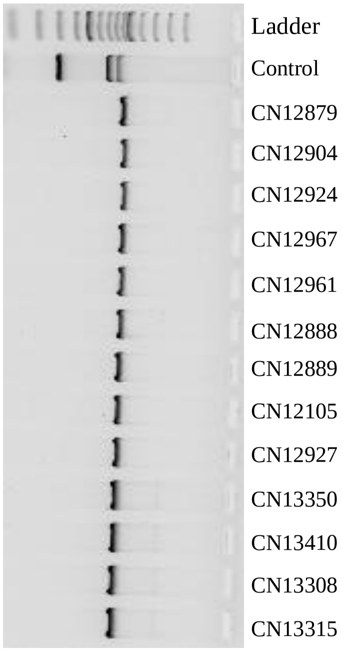
Ladder
Control
CN12879
CN12904
CN12924
CN12967
CN12961
CN12888
CN12889
CN12105
CN12927
CN13350
CN13410
CN13308
CN13315

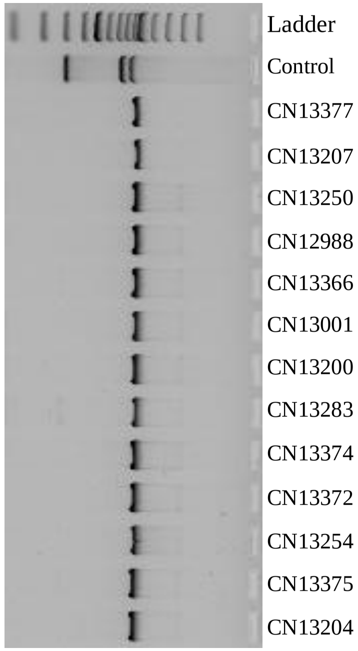
Ladder
Control
CN13377
CN13207
CN13250
CN12988
CN13366
CN13001
CN13200
CN13283
CN13374
CN13372
CN13254
CN13375
CN13204

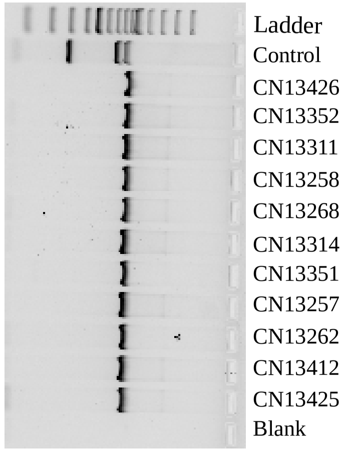
Ladder
Control
CN13426
CN13352
CN13311
CN13258
CN13268
CN13314
CN13351
CN13257
CN13262
CN13412
CN13425
Blank

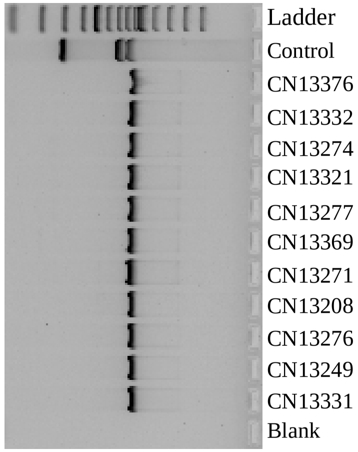
Ladder
Control
CN13376
CN13332
CN13274
CN13321
CN13277
CN13369
CN13271
CN13208
CN13276
CN13249
CN13331
Blank

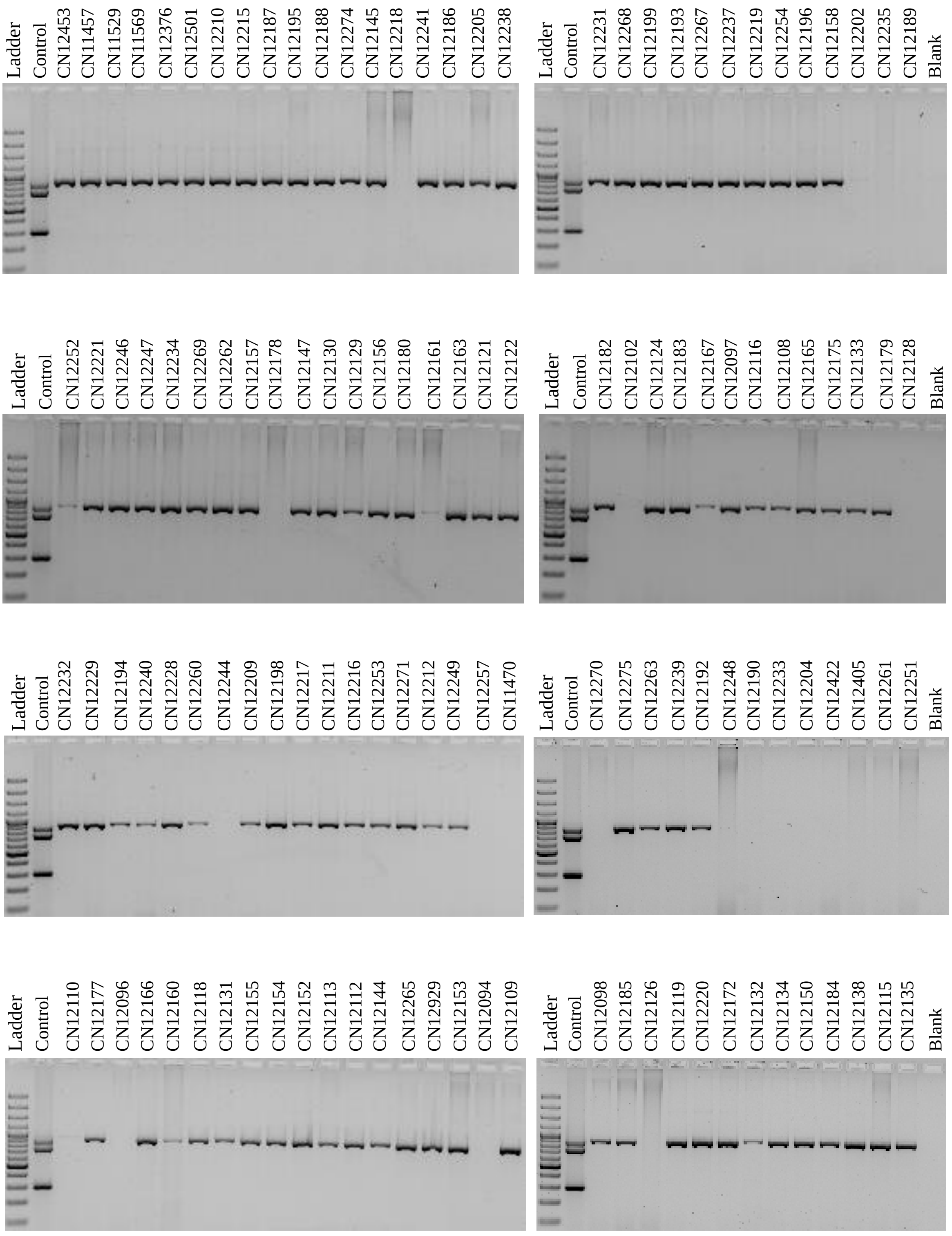
Ladder
Control
CN12453
CN11457
CN11529
CN11569
CN12376
CN12501
CN12210
CN12215
CN12187
CN12195
CN12188
CN12274
CN12145
CN12218
CN12241
CN12186
CN12205
CN12238
Ladder
Control
CN12231
CN12268
CN12199
CN12193
CN12267
CN12237
CN12219
CN12254
CN12196
CN12158
CN12202
CN12235
CN12189
Blank
Ladder
Control
CN12252
CN12221
CN12246
CN12247
CN12234
CN12269
CN12262
CN12157
CN12178
CN12147
CN12130
CN12129
CN12156
CN12180
CN12161
CN12163
CN12121
CN12122
Ladder
Control
CN12182
CN12102
CN12124
CN12183
CN12167
CN12097
CN12116
CN12108
CN12165
CN12175
CN12133
CN12179
CN12128
Blank
Ladder
Control
CN12232
CN12229
CN12194
CN12240
CN12228
CN12260
CN12244
CN12209
CN12198
CN12217
CN12211
CN12216
CN12253
CN12271
CN12212
CN12249
CN12257
CN11470
Ladder
Control
CN12270
CN12275
CN12263
CN12239
CN12192
CN12248
CN12190
CN12233
CN12204
CN12422
CN12405
CN12261
CN12251
Blank
Ladder
Control
CN12110
CN12177
CN12096
CN12166
CN12160
CN12118
CN12131
CN12155
CN12154
CN12152
CN12113
CN12112
CN12144
CN12265
CN12929
CN12153
CN12094
CN12109
Ladder
Control
CN12098
CN12185
CN12126
CN12119
CN12220
CN12172
CN12132
CN12134
CN12150
CN12184
CN12138
CN12115
CN12135
Blank

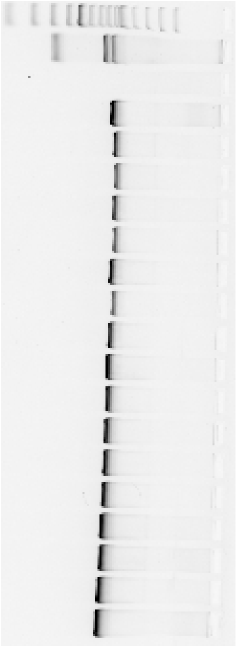
Ladder
Control
CN11513
CN12218
CN12202
CN12235
CN12189
CN12244
CN12257
CN11470
CN12270
CN12248
CN12190
CN12233
CN12204
CN12422
CN12405
CN12261
CN12251
CN12178

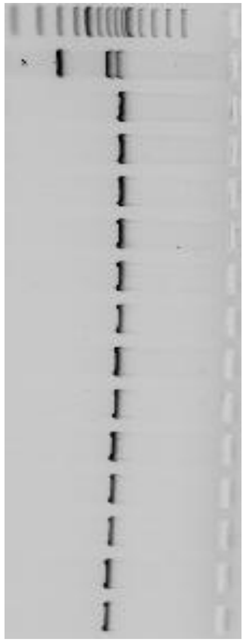
Ladder
Control
CN13275
CN13365
CN13205
CN13279
CN13318
CN13320
CN13246
CN12951
CN12952
CN12933
CN12890
CN12936
CN12114

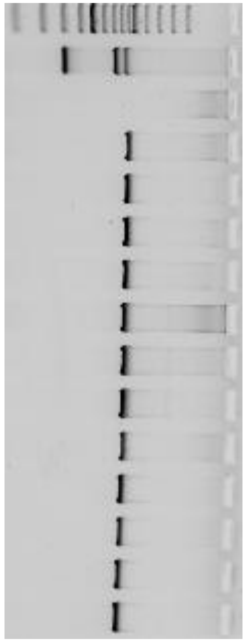
Ladder
Control
CN12400
CN12168
CN12162
CN12143
CN12169
CN12173
CN12120
CN12264
CN13012
CN13050
CN12978
CN13104
CN13051

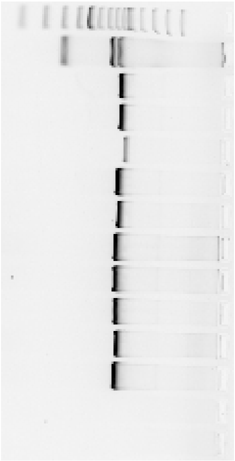
Ladder
Control
CN12102
CN12128
CN12110
CN12096
CN12141
CN12094
CN12126
CN12400
CN13055
CN12990
CN13059
Blank

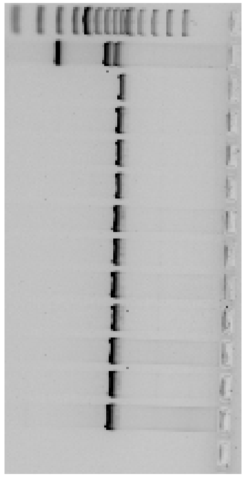
Ladder
Control
CN12100
CN12941
CN12962
CN12934
CN12949
CN12959
CN12948
CN12891
CN12935
CN12931
CN12103
Blank

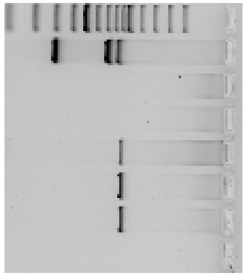
Ladder
Control
CN13055
CN12990
CN13135
CN12975
CN13098
Blank
